# Supplementary material for: The Impact of Domiciliary Dental Care and Oral Health Promotion in Nursing Homes of Older Adults: A Systematic Review
Source: Int J Environ Res Public Health. 2025 Apr 25;22(5):683. doi: 10.3390/ijerph22050683 (PMC12110923; doi:10.3390/ijerph22050683)
Supplement: Supplementary file 1 [file ijerph-22-00683-s001.zip › ijerph-3546469-supplementary.pdf]

Table S1. Database search strategy.

Database	Search strategy	Results
**PubMed**	((((((((aged[MeSH Terms]) OR (frail elderly[MeSH Terms])) OR (frail older adult[MeSH Terms]))) OR (aged[Title/Abstract])) OR ("frail elderly"[Title/Abstract])) OR ("frail older adult*"[Title/Abstract])) OR ("older adult"[Title/Abstract])) OR ("elderly"[Title/Abstract]) OR ("older patient"[Title/Abstract]) AND ((((((((dental care for aged[MeSH Terms]) OR (health services for aged[MeSH Terms])) OR (dental devices, home care[MeSH Terms])) OR (home care service*[MeSH Terms])) OR ("dental care for aged"[Title/Abstract])) OR ("health services for aged"[Title/Abstract])) OR ("home care services"[Title/Abstract])) OR ("home care"[Title/Abstract]) OR ("domiciliary care"[Title/Abstract]) OR ("home health care"[Title/Abstract]) OR ("domiciliary"[Title/Abstract]) AND ((((((((((((((((((((((((((((((((dental prostheses[MeSH Terms]) OR (dentures[MeSH Terms])) OR (dental caries[MeSH Terms])) OR (tooth diseases[MeSH Terms])) OR (periodontal diseases[MeSH Terms])) OR (oral manifestation[MeSH Terms])) OR (oral health[MeSH Terms])) OR (oral hygiene[MeSH Terms])) OR (xerostomia[MeSH Terms])) OR (mouth diseases[MeSH Terms])) OR (stomatognathic disease[MeSH Terms])) OR (dental plaque[MeSH Terms])) OR (toothbrushing[MeSH Terms])) OR (toothache[MeSH Terms])) OR ("dental prosthesis"[Title/Abstract])) OR ("denture"[Title/Abstract])) OR ("dental caries"[Title/Abstract])) OR ("periodontal disease*"[Title/Abstract])) OR ("oral manifestation*"[Title/Abstract])) OR ("oral health"[Title/Abstract])) OR ("oral hygiene"[Title/Abstract])) OR ("xerostomia"[Title/Abstract])) OR ("dental plaque"[Title/Abstract])) OR ("toothbrushing"[Title/Abstract])) OR ("carious lesion"[Title/Abstract])) OR ("oral patholog*"[Title/Abstract])) OR ("root carie*"[Title/Abstract])) OR ("dental carie*"[Title/Abstract])) OR ("dental disease"[Title/Abstract])) OR ("removable denture"[Title/Abstract])) OR ("plaque score"[Title/Abstract])) OR ("chewing difficult*"[Title/Abstract])) OR ("difficulty eating"[Title/Abstract])) OR ("oral disease"[Title/Abstract]) AND ((((((((health care cost[MeSH Terms]) OR (hospitalization[MeSH Terms]) OR (nutritional status[MeSH Terms])) OR (quality of life[MeSH Terms])) OR (chronic disease[MeSH Terms])) OR ("health cost"[Title/Abstract])) OR ("hospitalizarion"[Title/Abstract])) OR ("nutritional	**402**

Database	Search strategy	Results
	status"[Title/Abstract])) OR ("quality of life"[Title/Abstract])) OR (pneumonia[Title/Abstract])	
**Web of Science**	TS=(("aged" OR "frail elderly" OR "frail older adult" OR "older adult" OR "elderly adult" OR "older patient") AND (“dental care for aged” OR “health services for aged” OR “ home care dental service*” OR “home care service*” OR "home care" OR "domiciliary care" OR "home health care" OR "domiciliary") AND (“dental prostheses" OR "dentures" OR "dental caries" OR "tooth diseases" OR "periodontal diseases" OR "oral manifestation" OR "oral health" OR "oral hygiene" OR "xerostomia" OR "mouth diseases" OR "stomatognathic disease" OR "dental plaque" OR "toothbrushing" OR "toothache" OR "dental plaque" OR "carious lesion" OR "root carie*" OR "dental carie*" OR"dental disease" OR "removable denture" OR "plaque score" OR "chewing difficult*" OR "difficulty eating" OR "oral disease") AND ("health care cost" OR "hospitalization" OR "nutritional status" OR "quality of life" OR "chronic disease" OR "health cost" OR "pneumonia"))	**293**
**Scopus**	TITLE-ABS-KEY("aged" OR "frail elderly" OR "frail older adult" OR "older adult" OR "elderly adult" OR "older patient") AND TITLE-ABS-KEY ("dental care for aged" OR "health services for aged" OR "home care dental service*" OR "home care") AND ("dental prostheses" OR "dentures" OR "dental caries" OR "tooth diseases" OR "periodontal diseases" OR "oral manifestation" OR "oral health" OR "oral hygiene" OR "xerostomia" OR "mouth diseases" OR "stomatognathic disease" OR "dental plaque" OR "toothbrushing" OR "toothache" OR "dental plaque" OR "carious lesion" OR "root carie*" OR "dental carie*" OR "dental disease" OR "removable denture" OR "plaque score" OR "chewing difficult*" OR "difficulty eating" OR "oral disease") AND ("health care cost" OR "hospitalization" OR "nutritional status" OR "quality of life" OR "chronic disease" OR "health cost" OR "pneumonia")	**422**
**Lilacs**	("anciano" OR "idoso" OR "idoso fragilizado" OR "anciano frágil" OR "população idosa") AND ("Atención Odontológica para idosos" OR "Cuidado Dental para Ancianos" OR " Servicios de Salud para Ancianos" OR "Serviços de Saúde para	**0**

Database	Search strategy	Results
	Idosos" OR "Serviços de Assistência Domiciliar" OR "Servicios de Atención de Salud a Domicilio" OR "atendimento domiciliar") AND ("Prótese dentaria" OR "Prótesis Dental" OR "dentaduras" OR "doenças dentárias" OR "enfermedades dentales" OR "doença periodontal" OR "enfermedades periodontales" OR "manifestações bucais" OR "manifestaciones bucales" OR "saúde bucal" OR "salud bucal" OR "higiene bucal" OR "xerostomia" OR "placa dentária" OR "placa dental" escovação dentária" OR "cárie" OR "cepillado dental" OR " odontalgia") AND ("Custos de Cuidados de Saúde" OR "Costos de la Atención en Salud" OR "Hospitalización" OR "hospitalização" OR "Estado Nutricional" OR "qualidade de vida" OR "calidad de vida" OR "doença crônica" OR "enfermedad crônica")"	
**Embase**	('older adult*' OR 'older' OR 'aged'/exp OR 'aged' OR 'older people'/exp OR 'older people' OR 'older patient*') AND ('dentistry for aged' OR 'dental care for elderly' OR 'home care'/exp OR 'dental device'/exp OR 'dental care team'/exp) AND ('periodontal health'/exp OR 'periodontal health' OR 'carious lesion*' OR 'dental cavities'/exp OR 'dental cavities' OR 'oral pathologies' OR 'dental caries'/exp OR 'gingival blending' OR 'tooth disease'/exp OR 'removable denture' OR 'oral habit*' OR 'number of teeth' OR 'denture hygiene' OR 'denture/exp' OR 'denture' OR 'edentulous jaw'/exp OR 'inflammation of oral mucosa' OR 'candidiasis'/exp OR 'candidiasis' OR 'plaque'/exp OR 'plaque' OR 'plaque score'/exp OR 'plaque score' OR 'chewing difficult' OR 'dysphagia/exp' OR 'prosthesis'/exp OR 'tooth pain'/exp OR 'mouth disease'/exp) AND ('medical care cost' OR 'health cost' OR 'treatment cost'/exp OR 'treatment cost' OR 'oral health-related quality of life' OR 'quality of life'/exp OR 'hospitalization'/exp OR 'hospitalization' OR 'respiratory'/exp OR 'respiratory' OR 'pneumonia'/exp OR 'pneumonia' OR 'nutritional')	**35**
**Cochrane**	#1 (Aged) OR (Frail Elderly)<br>#2 (Dental Care for Aged) OR (Health Services for the Aged) OR (Home Care Services) OR (Dental Devices) OR (Home Care)<br>#3 (Dental Prosthesis) OR (Dentures) OR (Dental Caries) OR (Tooth Diseases) OR (Periodontal Diseases) OR (Oral Manifestations) OR (Oral Health) OR (Oral	**1702**

Database	Search strategy	Results
	hygiene) OR (Xerostomia) OR (Mouth Diseases) OR (Stomatognathic Diseases) OR (Dental plaque) OR (Toothbrushing) OR (Toothache)<br>#4 (Health Care Costs) OR (Hospitalization) OR (Nutritional Status) OR (Quality of Life) OR (Chronic Disease)	
**Google Scholar**	Older adult* AND Home Health Care AND Oral Pathologies AND quality of life	**4**
**Proquest**	Older adult* AND Home Health Care	**0**
